# Functional bottlenecks for generation of HIV-1 intersubtype *Env* recombinants

**DOI:** 10.1186/s12977-015-0170-8

**Published:** 2015-05-23

**Authors:** Bernard S. Bagaya, José F. Vega, Meijuan Tian, Gabrielle C. Nickel, Yuejin Li, Kendall C. Krebs, Eric J. Arts, Yong Gao

**Affiliations:** Department of Molecular Biology and Microbiology, School of Medicine, Case Western Reserve University, 10900 Euclid Ave, Cleveland, OH 44106 USA; Division of Infectious Diseases, Department of Medicine, Case Western Reserve University, 10900 Euclid Ave, Cleveland, OH 44106 USA; Department of Microbiology and Immunology, Schulich School of Medicine and Dentistry, Western University, London, ON N6A 5C1 Canada

## Abstract

**Background:**

Intersubtype recombination is a powerful driving force for HIV evolution, impacting both HIV-1 diversity within an infected individual and within the global epidemic. This study examines if viral protein function/fitness is the major constraint shaping selection of recombination hotspots in replication-competent HIV-1 progeny. A better understanding of the interplay between viral protein structure-function and recombination may provide insights into both vaccine design and drug development.

**Results:**

In vitro HIV-1 dual infections were used to recombine subtypes A and D isolates and examine breakpoints in the Env glycoproteins. The entire *env* genes of 21 A/D recombinants with breakpoints in gp120 were non-functional when cloned into the laboratory strain, NL4-3. Likewise, cloning of A/D gp120 coding regions also produced dead viruses with non-functional Envs. 4/9 replication-competent viruses with functional Env’s were obtained when just the V1-V5 regions of these same A/D recombinants (i.e. same A/D breakpoints as above) were cloned into NL4-3.

**Conclusion:**

These findings on functional A/D Env recombinants combined with structural models of Env suggest a conserved interplay between the C1 domain with C5 domain of gp120 and extracellular domain of gp41. Models also reveal a co-evolution within C1, C5, and ecto-gp41 domains which might explain the paucity of intersubtype recombination in the gp120 V1-V5 regions, despite their hypervariability. At least HIV-1 A/D intersubtype recombination in gp120 may result in a C1 from one subtype incompatible with a C5/gp41 from another subtype.

## Background

A major obstacle for HIV treatment and vaccine development is virus diversity which continues to increase due to its high mutation rate and recombination [1–6]. Intersubtype recombination is shaping HIV evolution by establishing unique and stable circulating recombinant forms (URFs and CRFs) in various regional epidemics [7–14], by contributing to the rapid emergence of multi-drug resistance [15, 16] and immune escape [17, 18], and by rescuing HIV-1 from catastrophic mutations via negative epistasis [19]. In this study, we have explored the functional constraints that limit intersubtype recombination in the HIV-1 *env* gene. These mechanistic studies on HIV-1 recombination can provide valuable insight into chimeric *env* cloning and production, the basis for many HIV-1 vaccine designs. Likewise, understanding the limitations in functional complementation within the *env* coding region can be advantageous as a therapeutic target and for drug design.

The HIV-1 envelope is a glycoprotein trimeric complex found on the viral surface, embedded in the membrane, and composed of the gp120 subunit spikes in a non-covalent interaction with the gp41 harboring the transmembrane domain. Each gp120–gp41 subunit is derived from the proteolytic processing of the envelope precursor gp160 in the Golgi complex [20, 21]. The envelope trimer collectively coordinates entry of HIV-1 into susceptible cells. The gp120 glycoprotein is subdivided into a conserved core derived from five conserved subdomains (C1–C5) interspersed by five hypervariable, glycosylated loops (V1-V5) [22, 23]. The C4 region of gp120 mediates binding to the host CD4 molecule, inducing a conformational change, and promoting interaction between gp120 C2 and V3 regions with the N terminus and 2^nd^ extracellular loop of CCR5 (or CXCR4). The gp41 senses the conformational changes in gp120 and undergoes a radical structural refolding culminating in the fusion of viral and host cell membranes [24, 25]. The second exons of *tat* and *rev* overlap with the gp41 coding region of HIV-1 *env,* and must be correctly spliced to join the first exons to produce functional Tat and Rev proteins which are two essential viral regulatory factors for HIV gene expression [26, 27]*.* Thus, any modifications at the gp120/gp41 coding interface due to the intersubtype recombination, could alter the correct splicing of the *tat* and *rev* mRNA and possibly disrupt the function of Tat and Rev proteins. However, significant intersubtype sequence variability in both *tat* and *rev* sequences exists, and even in the same subtype Tat and Rev continue to evolve under selection pressure [28, 29].

We have previously examined the emergence and selection of intersubtype HIV-1 recombinants in single cycle systems involving replication defective viruses and in dual infection studies [1, 2, 30, 31]. With increasing selection for replication competent viruses using various in vitro systems, we observed a re-distribution of *env* recombination sites within the gp120 coding sequence (no selection) to breakpoints primarily located in the gp120/gp41 interface (selection for fully functional Env’s). Intersubtype recombination within the HIV-1 gp120 coding region could impact complementation between the subdomains of gp120 and produce Env glycoproteins that are not properly expressed, modified, or transported to the cell surface. Intersubtype recombinants with breakpoints in gp120 also provide a unique circumstance to study intermolecular interactions within the HIV-1 envelope. Even when incorporated into a new virus particle, such chimeric Envs may be defective for subsequent host cell entry. Likewise, a recombination breakpoint in the gp120 coding region of *env* could also impact the function of the accessory proteins, Rev and Tat. We have characterized a set of HIV-1 intersubtype A and D Env recombinants with breakpoints in the gp120 and gp41 coding region and have shown that A/D breakpoints between the C1 and C5 domains of gp120 result in non-functional, replication-defective Env glycoproteins. This study now provides strong evidence of co-evolution and direct interplay between the C1, C5, and gp41 domains of the Env glycoproteins, necessary for host cell entry and viral infectivity.

## Results

### Generation of Intersubtype *envA/D* recombinants

**Fig. 1:**
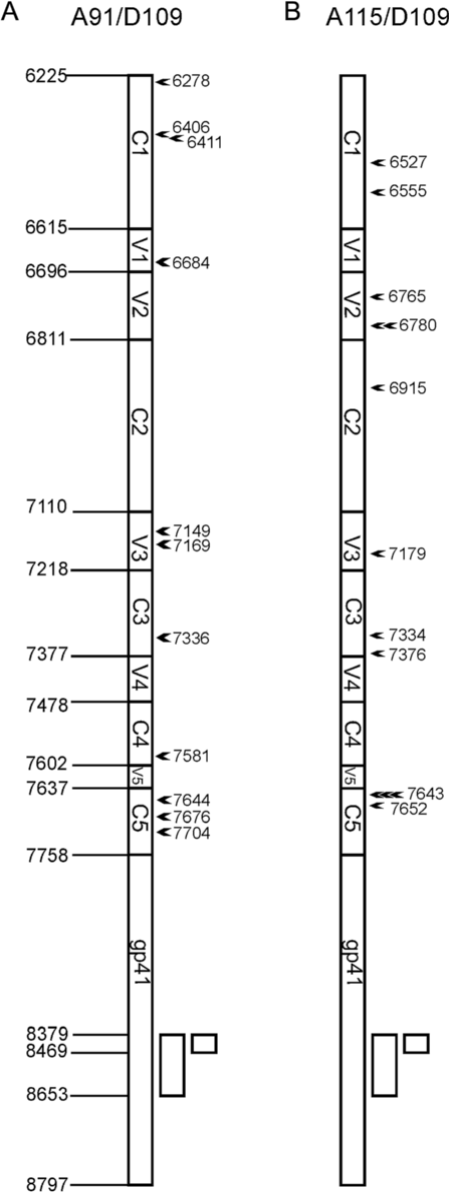
Distribution of recombination breakpoints in *gp120* region. Clonal *gp120* recombinant sequences were aligned with parental strains and the recombination breakpoints were determined using similarity plots constructed in SimPlot sequence analysis software. Distribution of breakpoint in *gp120* region for A91/D109 and A115/D109 dual infection sets is indicated in (**a**) and (**b**) respectively

We have previously described dual infection methods to produce HIV-1 *env* recombinants [32]. Even though PBMCs are more relevant in generating recombinants similar in HIV patients, based on our previous experience, the infection efficiency of HIV in PBMCs is much lower than in U87.CD4.CCR5 cell line resulting in very low levels of recombinant genomes, and the sites of intersubtype recombination was similar in U87 cells and PBMCs [1, 2, 30, 31]. In this study, subtype A (A91 and A115) and D (D109) HIV-1 isolates were used to co-infect U87.CD4.CCR5 cells. Subtypes A and D co-circulate in Kenya and Uganda and frequently recombine to generate A/D URFs, isolated in HIV-infected individuals. We cloned and sequenced 88 *envA/D* recombinants from dual infections of the A91 + D109 and of the A115 + D109 HIV-1 isolates. Of 88 *envA/D* recombinants, 28 had recombination breakpoints within *gp120* while the remaining breakpoints mapped to the highly conserved, transmembrane coding region of *gp41*. Seven A/D *gp120* recombinants were excluded based on premature stop codons or frameshift in the envelope open reading frame (ORF) or duplicates with the same unique breakpoint. The remaining 21 unique A/D recombinants with breakpoints in *gp120*, 11 A91/D109 (Fig. 1a) and 10 A115/D109 (Fig. 1b), were used to investigate the impact of gp120 recombination on Env function. Except bk6780 (breakpoint at position 6780) and bk7643, all of the other breakpoints identified in the gp120 region were single (Fig. 1).

### Full length HIV-1 A/D recombinant envelopes with breakpoints in *gp120* were non-functional when cloned into NL4-3 backbone

**Fig. 2:**
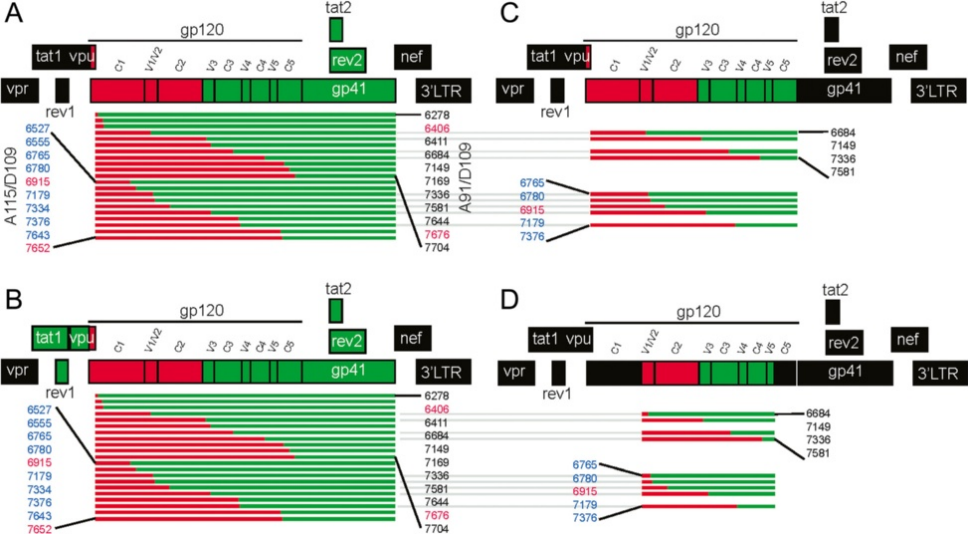
Systematic strategies for cloning *envA/D* recombinant sequences. (**a**) Full *envA/D* recombinants were cloned into a plasmid containing a near full length HIV-1 NL4-3 backbone; (**b**) plasmids cloned in panel A were modified by insertion of *tat1*, *rev1* and *vpu* from isolate D109 to match the corresponding *tat2* and *rev2* sequences; (**c**) selected *gp120* recombinants from panel B were cloned such that NL4-3 gp41 was unaltered; (**d**) only *V1-V5* of recombinant *gp120* sequences was cloned in order to preserve the consistency between C1, C5, and gp41 domains. Subtype A sequences (A115 and A91) are denoted by red lines, and subtype D (D109) sequences are denoted by green lines

We and others have previously described intersubtype recombination “hotspots” in the *env* gene derived from single-cycle assays, dual infection systems, and recombination enrichment systems involving siRNA selection [1, 2, 30–33]. With increased selection for replication-competent viruses, there was limited intersubtype recombination within the gp120 coding regions and increased detection of breakpoints in the gp41 coding regions. In this study, we examined the function of Env when intersubtype recombination was identified in the gp120 coding sequence. We first cloned full length *env* with A/D gp120 breakpoints (aka *env-gp120* A/D) into pREC_nfl_HIV∆env/URA3 plasmid (containing an HIV-1 NL4-3 near full length genome except 5′LTR sequence as a backbone) (Fig. 2a) using a yeast-based recombination method [34]. The cloned *env-gp120* A/D recombinants in the NL4-3 backbone were tested for their ability to mediate cell fusion using the Veritrop assay (a surrogate of virus entry) as previously described [35]. These env–gp120 A/D vectors were also co-transfected with a complementing vector [34] to measure virus production and subsequent multiple cycle replication.

**Fig. 3:**
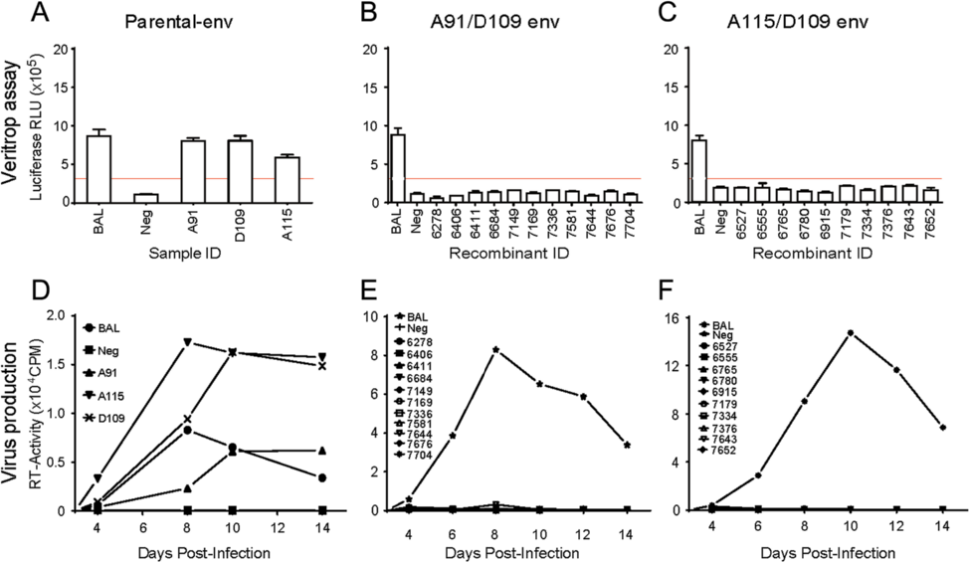
Function assay of HIV-1 envelope recombinants with breakpoints in *gp120* when entirely cloned into NL4-3 backbone. Parental HIV-1 isolates A91, A115 and D109 *envs*, and *envA/D* recombinants of A91/D109 and A115/D109 with breakpoints in *gp120* region were cloned with the entire *env* sequences into NL4-3. Veritrop assay (fusion/function assay) and virus propagation results of parental envelopes (**a** and **d**), *env* recombinants from A91/D109 pair (**b** and **e**) and from A115/D109 set (**c** and **f**) are plotted

All of the parental A91, A115 and D109 HIV-1 *env* genes in pREC_nfl_HIV_NL4-3_ mediated robust cell-to-cell fusion when tested by using the Veritrop assay [35] (Fig. 3a). However, the 21 *env-gp120* A/D recombinants (11 A91/D109 and 10 A115/D109) in pREC_nfl_HIV_NL4-3_ did not mediate cell-to-cell fusion (Fig. 3b and c). A similar outcome was observed with the respective recombinant viruses. Spreading virus infections were observed with the NL4-3 containing the parental A91, A115 and D109 HIV-1 *env* genes (Fig. 3d) whereas the viruses with the *env-gp120* A/D recombinants were replication defective (Fig. 3e and f).

### Impact of chimeric Tat/Rev proteins on replication of A/D recombinant viruses

**Fig. 4:**
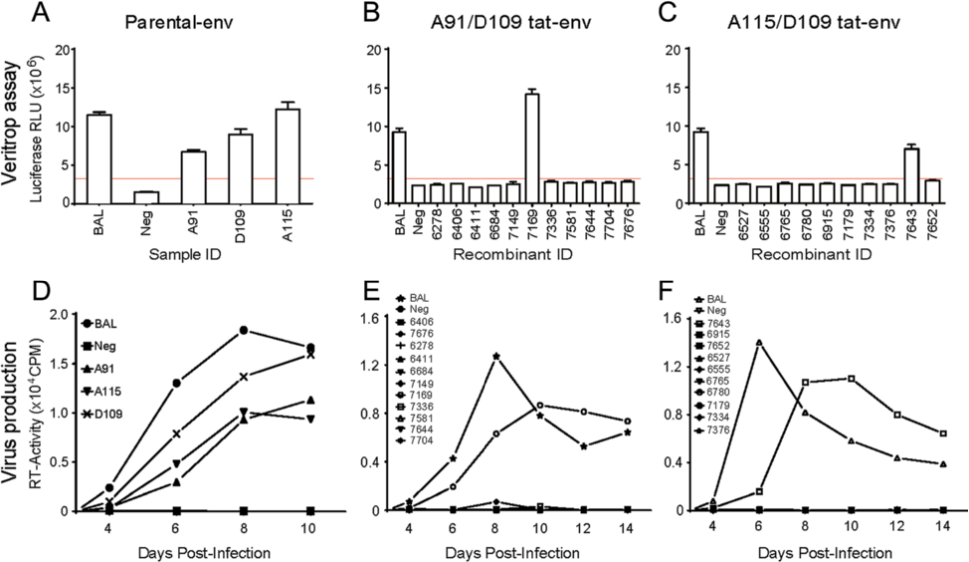
Influence of discordance in *tat/rev* exon 1 and 2 on HIV-1 intersubtype *envA/D* recombinant envelopes. The *tat* exon 1 sequence from isolate D109 was cloned into the non-functional plasmids containing entire *envs* from HIV-1 A91, A115 and D109 parental virus (**a** and **d**), *env* recombinants from A91/D109 pair (**b** and **e**) and from A115/D109 set (**c** and **f**) which were subjected to Veritrop assay and virus replication

HIV-1 Tat and Rev proteins are translated from two exons. The first exons of *tat* and *rev* are located upstream of the env gp120 coding region, while *tat*2 and *rev*2 exons are found in the gp41 coding region. In the pREC_nfl_HIV constructs containing the *env-gp120* A/D recombinants, *tat1/rev1* exons are of NL4-3 sequence and spliced to *tat2/rev2* exons of D109 sequence resulting in chimeric Rev and Tat proteins. Thus, defective pREC_nfl_HIV constructs with *env-gp120* A/D may be the consequence of non-functional, chimeric Tat/Rev accessory proteins rather than the direct consequence of a chimeric A/D Env glycoprotein. To rule out this possibility, we replaced the NL4-3 *tat1* and *rev1* exons in the backbone with the corresponding sequence from isolate D109 such that both *tat/rev* exons would be derived from the same subtype D HIV-1 isolate in the *env-gp120* A/D recombinants (see Methods) (Fig. 2b). Two of the 21 *env-gp120* A/D recombinants, one from the set of A91/D109 (Fig. 4b and e) and one from A115/D109 (Fig. 4c and f), were functional upon introduction of the concordant D109 *tat1* and *rev1* exons. This result suggests that in some instances, chimeric A/D Tat and Rev proteins can restrict or reduce HIV-1 replication of some intersubtype recombinants with breakpoints appearing in the gp120 coding region of *env*. However, the majority of *env-gp120* A/D recombinants remained non-functional indicating that sequence concordance in the first and second exon of *tat/rev* is not the primary cause of this restriction. Please note that *vpu* in the cloned HIV-1 viral genome is also chimeric (B/A). However, the constructs with the primary *envs* containing the same *vpu* produced replicative viruses (Fig. 3a and d) suggesting that it has no significant influence in the replication capacity.

### Impact of A/D gp120 coding region on replication of A/D recombinant viruses

The gp120 region of the HIV-1 envelope is responsible for CD4 binding, coreceptor recognition, immunogenicity, immune evasion, interactions with gp41, and coordinating the host cell entry process. Thus, intersubtype recombination in gp120 can result in a significant shift in genetic differences and could result in a non-functional envelope on the viral surface. By cloning just the A/D gp120 region into the NL4-3 backbone, there was no disruption of another HIV-1 coding region and we could retain *tat* and *rev* genes from the NL4-3 backbone. Thus, we could examine the impact of intersubtype recombination sites on gp120 function (in association with other interacting proteins, e.g. gp41). On the other hand, based on possible interactions between the N and C-terminus of gp120 in the crystal structure [36–38], we suspect that the function of A/D recombinants with breakpoints in V1-V5 domains might be maintained if retaining the NL4-3 C1 and C5 domains. Cloning the parental A91, A115 and D109 V1-V5 region into the NL4-3 backbone resulted in functional Env and replication competent virus suggesting again that this subtype B NL4-3 virus could accommodate a diverse chimeric Env glycoprotein.

**Fig. 5:**
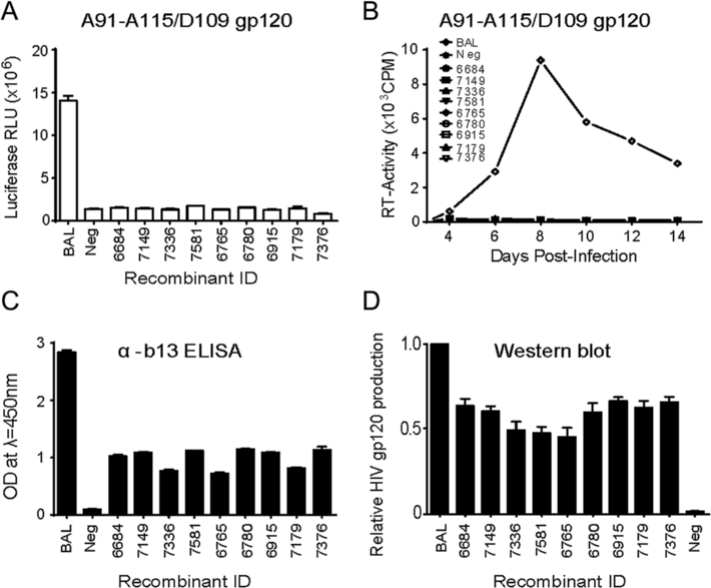
Cloning of only *gp120* sequences of intersubtype *envA/D* recombinants into NL4-3 backbone and the resultant envelope function assay. Intersubtype A/D recombinant *gp120* genes were cloned, leaving gp41 of NL4-3 intact. (**a**) The plasmids containing recombinant *gp120* sequences were tested by Veritrop assay for functionality and (**b**) for replication-competent virus production. Pseudoviruses produced from the plasmids were tested by ELISA (**c**) and Western blot (**d**) for the production of envelope expression

**Fig. 6:**
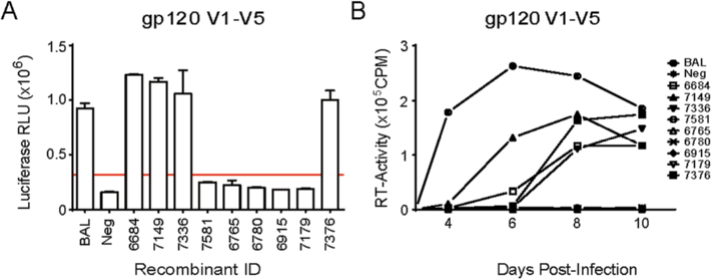
Cloning of only *V1-V5* domains of *envA/D* recombinants into NL4-3 backbone and the resultant envelope function assay. The *V1-V5* domains of 9 *envA/D* recombinants were cloned into NL4-3 backbone, and were tested for their functionality by Veritrop assay (**a**) and for their ability to produce replication-competent viruses (**b**)

For these analyses, we cloned either the entire gp120 coding region or only V1-V5 region of 9 A/D *gp120* recombinants with breakpoints in *gp120* (bk6684, bk7149, bk7336, and bk7581 from A91/D109, and bk6765, bk6780, bk6915, bk7179, and bk7376 from A115/D109), all containing breakpoints within V1-V5 regions into NL4-3 backbone (Fig. 2c and 2d). We excluded the analyses of *gp120* A/D recombinants which contained breakpoints either in C1 or C5 region (i.e. bk6278, bk4406, bk6411, bk7169, bk7644, bk7676, bk7704, bk6527, bk6555, bk6915, bk7334, bk7643, and bk7652). The cloning of the entire gp120 coding regions of these 9 A/D *gp120* recombinants resulted in none of functional Env glycoprotein by Veritrop or replication defective virus (Fig. 5a and b). Please note that all 9 A/D gp120 coding regions cloned into NL4-3 backbone resulted in production of recombinant Env glycoproteins on the viral surface as demonstrated by EIA using plates coated with anti-gp120 antibody B13 (Fig. 5c), as well as Western blot (Fig. 5d). Interestingly, 4 out of 9 A/D V1-V5 regions cloned into an NL4-3 backbone (in between the C1 and C5 regions of NL4-3) resulted in functional Env glycoproteins and replication competent viruses (Fig. 6a and b). Although bk6684 (breakpoint at V1/V2 region), bk7149 (V3), bk7336 (C3), and bk7376 (C3) could not replicate or produce functional Env when cloned as a whole Env or gp120, the cloning of V1-V5 region of these A/D recombinants successfully rescued their function (Fig. 6a and b).

### Co-evolution and structural interactions between gp120 C1, C5, and ecto-gp41 domains

Our studies on intersubtype recombination in *env* as well as sequence analyses of patient-derived URFs and CRFs revealed that breakpoints are rarely observed in the V1-V5 gp120 coding region. There are only 4 listed A/D CRFs (and 1 A1DG), and 2 of these are A/D in Env. CRF35_A1D Env is A1, with a small region in the C4/C5 region being subtype D, while CRF50_A1D has breakpoints between C1 and C3 (http://www.hiv.lanl.gov). Again, when Env function is required, recombination events in *env* tend to cluster in the leader-C1 region of gp120 or in the transmembrane domain of gp41 [30, 31]. In the absence of functional selection, intersubtype recombination is quite prevalent in the other conserved regions C2, C3, and C4 and still evident in the hypervariable sequences. Previous studies indicate that cross-overs/breakpoints can occur anywhere on the template during reverse transcription but are more frequent at regions of higher sequence conservation [30, 31].

**Fig. 7:**
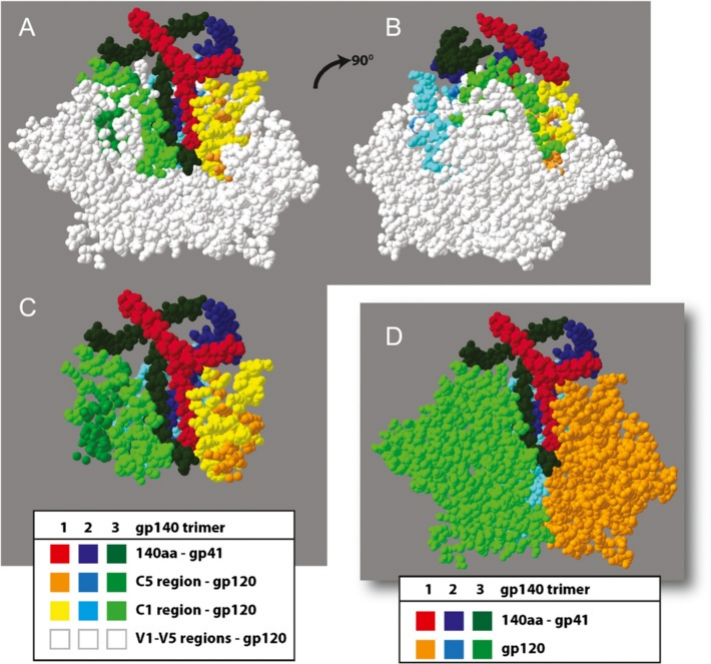
Model of the C1/C5/ecto-gp41 core in the crystal structure of the HIV-1 gp140 trimer. This figure is derived from the recent 4.7 Å crystal structure of a soluble, cleaved BG505 SOSIP.664 gp140 trimer in complex with a potent bnAb, PGT122 [37] (PDB - 4NCO). The interaction between the gp120 C1, C5, and ecto-gp41 domains is highlighted. For this model, the bnAb PGT122 is hidden, viral membrane would be on top, and CD4/CCR5 would engage at the bottom of each view. The Env trimer “core” of the C1, C5, and ecto-gp41 of each gp140 subunit is shown in panel (**a**), turned 90^O^ in (**b**), and stripped of all other gp120 residues in panel (**c**). Finally, each gp140 subunit of the trimer is shown in panel (**d**)

The absence of functional intersubtype recombinants with breakpoints between the C1 and C5 domains of HIV-1 gp120 may be associated with co-evolving interactions between the linear ends of the gp120 and with the ecto domain of gp41. Galli A, et al. studied the recombination in pol region between two subtype B sequences (B/B) and between one subtype B and one subtype F sequence (B/F), and found the evidence for the evolved co-adapted sites in variants from different subtypes [39]. Numerous studies have mapped possible interaction between the gp120 C1 and C5 domains with the ecto domain of gp41, which is now supported by a recent 4.7 Å crystal structure of a soluble, cleaved BG505 SOSIP.664 gp140 trimer in complex with a potent broadly neutralizing antibody (bnAb), PGT122, and recent 5.8 Å cryo-electron microscopy re-construction of the Env trimer in complex with a CD4 binding site bnAb, PGV04 [36–38, 40–44]. In Fig. 7, we have utilized the crystal structure coordinates of the Env trimer (PDB - 4NCO) to highlight the interaction between the gp120 C1, C5, and ecto-gp41 domains (residues 1–126). For this model, the bnAb PGT122 is hidden, viral membrane would be on top, and CD4/CCR5 would engage at the bottom of each view (Fig. 7a). The Env trimer “core” containing the close association between C1, C5, and ecto-gp41 domains of each gp120 subunit is shown by turning the structure 90° clockwise (Fig. 7b) and by being stripped of all other gp120 residues (Fig. 7c). These trimer domains are less exposed and more encased by the remaining gp120 regions (Fig. 7d).

**Fig. 8:**
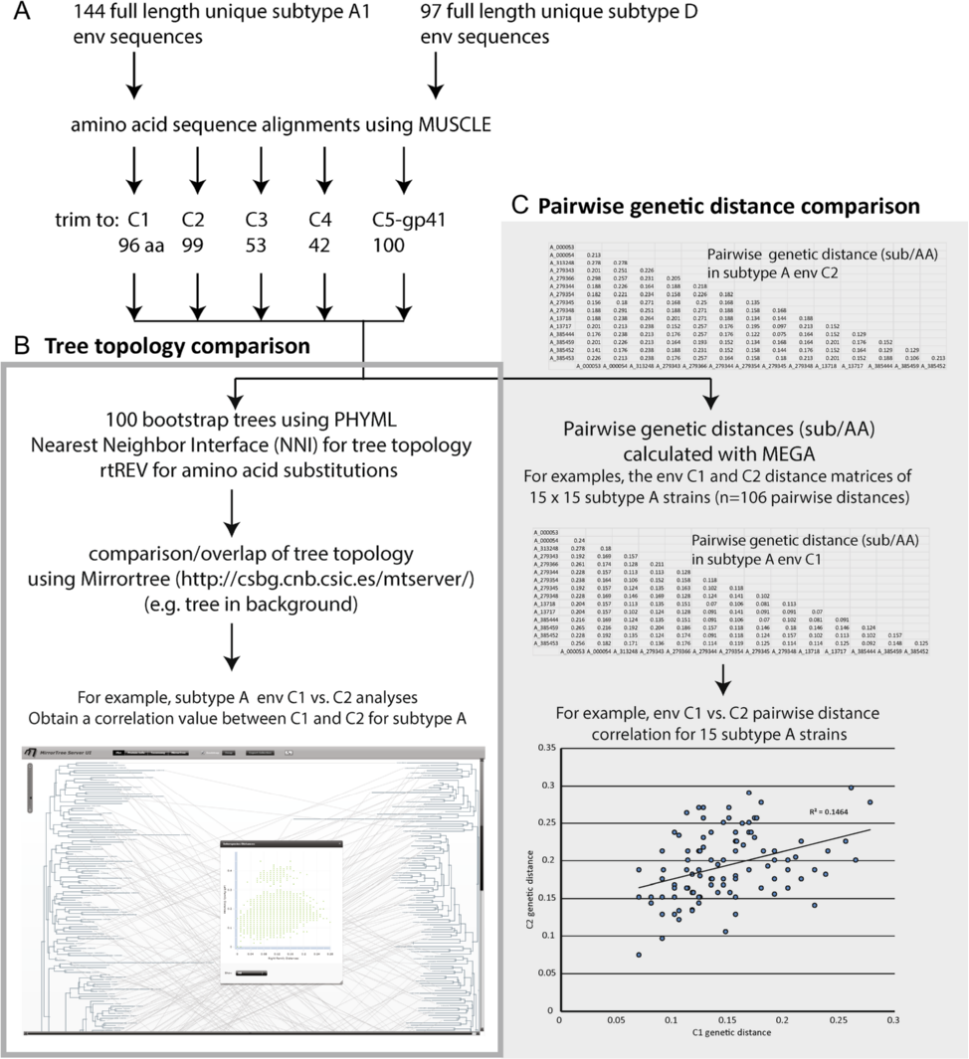
Schematics of Phylogenetic/coevolution analyses. (**a**) Full length subtype A1 and subtype D envelope amino acid sequences downloaded from the Los Alamos National Laboratory HIV sequence database were filtered and resulted in data sets containing 144 and 97 unique sequences of subtype A1 and subtype D, respectively. Subtype specific alignments were performed using MUSCLE and then trimmed to the positions containing the C1–C5 regions and the ecto-gp41 domain. (**b**) Maximum likelihood trees were generated for each region using the algorithms implemented in PHYML with 100 bootstrap replicates, and the tree topology search was performed using Nearest Neighbor Interchanges (NNIs). The phylogeny of the regions was estimated using the rtREV model of amino acid substitution. The resulting maximum likelihood trees were used as input to the Mirrortree web server. (**c**) Pairwise distance matrices of each region were built with MEGA, which used a JTT matrix-based model and a uniform substitution rate among sites, to calculate the number of amino acid substitutions per site/residue from between each sequence in the alignment. Correlation between each conserved region in gp120 or the gp41 domain were then measured by Pearson’s product-moment correlation coefficient, and significance estimated with Fisher’s exact test

**Fig. 9:**
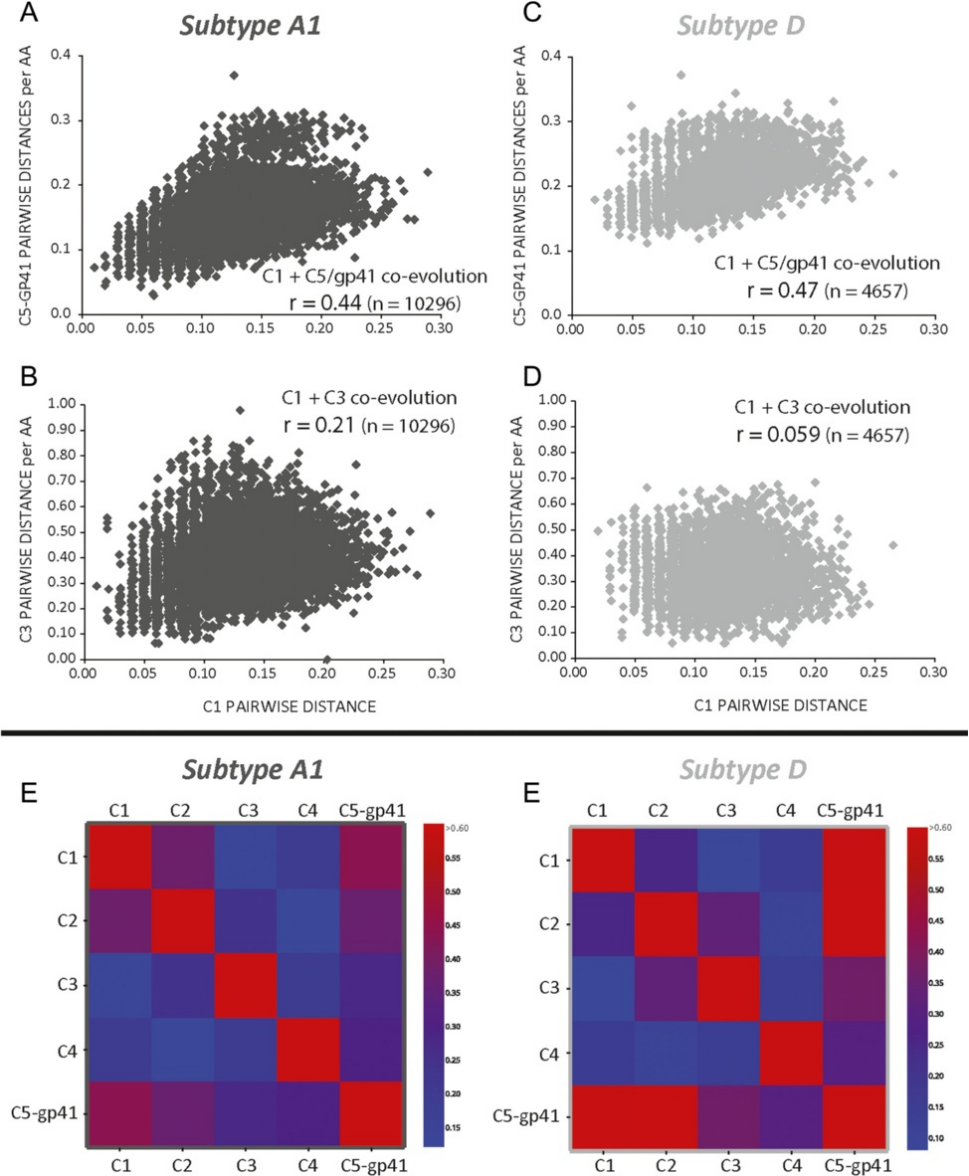
Co-evolution of the C1/C5/ecto-gp41 domains in HIV-1 subtype A and D isolates. Scatter plots demonstrating the correlation between the pairwise distance matrices of viral isolates were built from multiple sequence alignments of unique subtype A1 and subtype D *env* sequences (n = 10,296, (**a**) and (**b**); n = 4657 (**c**) and (**d**), respectively). Pairwise comparisons were performed with all pairs of conserved C1, C2, C3, C4, and C5-gp41 regions but only the comparison of C1 + C5/gp41 (**a** and **c**) and of C1 + C3 (**b** and **d**) are presented in this figure. Pearson product moment correlations are shown for each panel but due to high number of sample sequences, p values were highly significant (<0.0001) for all paired analyses suggesting some level of co-evolution throughout the protein as expected. The heatmaps in (**e**) and (**f**) reflect the tree correlation coefficients derived from a similarity comparison of the maximum likelihood derived trees of the multiple sequence alignments of the subtype specific conserved regions. The correlation coefficients between C1 and C5-gp41 demonstrate the strongest similarity of tree topology among the regions compared

Analyses of the Env trimers suggest a close interaction between C1, C5, and ecto-gp41 domains despite the ~340 amino acids separating this N and C terminus in the linear sequence. Thus, lack of “functional” intersubtype recombinations with breakpoints between the C1 and C5 domains may be related to the inability of the subtype A C1 domain to interact with the subtype D C5 and ecto-gp41 domains. If this is the case, we should observe co-evolution between the C1 and C5/ecto-gp41 domains within a subtype. As described in the Methods and Fig. 8, we constructed separate maximum likelihood trees on the coding regions of C1, C2, C3, C4, C5/ecto-gp41 domains using 144 and 97 unique subtype A1 and D Env sequences (100 bootstrap replicates for each), and using the rtREV [45] model for phylogenetic inference of amino acid substitutions. We then compared the tree topology of each matched terminal taxa in the tree for all pairs of conserved Env regions (e.g. C1 + C2, C1 + C3, C1 + C4, C1 + C5/ecto-gp41, etc.) using Mirrortree web server [46] which was developed to predict functional relationships between protein families. In other words, relative genetic distances between all 144 branches of the subtype A1 tree of C1 domain are compared to that of the subtype A1 tree of C2 domain. Heatmaps demonstrating the correlation between each paired domain in Env were produced with Plot (https://plot.ly). To simplify, the strongest co-evolution between two conserved Env domains would have the best overlay between tree topology of those conserved regions and show the strongest correlation. With both subtype A1 and D, the C1 domain shows the strongest co-evolution with the C5/ecto-gp41 domain (r = 0.44 and 0.47, respectively) (Fig. 9a and c) comparing with other regions, e.g. C3 domain (Fig. 9b, and d). Interestingly, linear order of the conserved regions does not exhibit consistent evidence of co-evolution along the length of the gene, indicating that the proximity of the regions to each other was not the driving factor of covariance (i.e. co-evolution). For example, C1 is separated from C2 by ~66 amino acids (V1/V2) and shows a moderate pattern of covariance (r = 0.26–0.39), while C3 and C4, who are separated by a much shorter distance of ~11 amino acids, show a weak pattern of covariance (r = 0.07–0.22).

To further predict protein co-evolution, we used the methods developed in Pazos and Valencia [47], which are similar to the methods implemented in mirrortree with the exception that phylogeny of the viruses is not taken into consideration since correlation is based solely on the underlying distance matrices. The Jones-Taylor-Thornton (JTT) matrix-based model [48] uses a uniform substitution rate among sites to calculate the number of amino acid substitutions per site between each sequence in the alignment. The pairwise distances were calculated for paired conserved region (C1, C2, C3, C4, and C5-gp41) of every virus in the multiple sequences, generating 10,296 and 4656 data points for subtype A1 and D viruses. Pearson’s product-moment correlation coefficient was calculated to measure the relationship between the distances paired conserved regions for these viruses. As in the analysis of the maximum likelihood tree topology described above, the strongest correlations existed between C1 and C5-gp41 (Fig. 9e and f).

## Discussion

We and others have mapped intersubtype recombination in the *env* gene using various HIV-based systems. The frequency and site of intersubtype recombinations is governed by sequence conservation, RNA secondary structures, and selective pressure for subsequent virus replication [1, 30, 33, 49, 50]. However, few studies [1, 33, 51] have characterized the function of these intersubtype recombinant genomes generated in vitro. Our laboratory has developed several systems to understand the mechanisms involved in HIV-1 recombination. Early studies explored the generation of intersubtype HIV-1 recombinants in the *env* gene by dually infected susceptible cell cultures with HIV-1 subtypes that co-circulated in regional epidemics [31, 32]. Dual infection generally produces intersubtype recombinant viruses at low frequency that are defective or that cannot compete with the parental viruses. To enrich for “functional” recombinants, we utilized HIV isolate-specific siRNAs that prevented outgrowth of parental viruses while permitting replication of intersubtype recombinants with breakpoints that “recombined out” the siRNA target sequences [2]. With both studies, the preliminary evidence still suggested that intersubtype breakpoints in gp120 led to non-functional envelopes and defective viruses. However, we did not employ a systematic approach to investigate the impact of intersubtype recombination in the *env* gene on function and support of HIV-1 replication. The mechanism of HIV-1 intersubtype recombination during reverse transcription does not, of course, account for the functionality of the resulting genomes, which is largely controlled by the minus strand DNA invasion onto another HIV-1 RNA genome during pausing of reverse transcriptase at homopolymeric tracts or RNA secondary structures. Again, these strand transfer events occur at increased frequency in regions of high sequence conservation [1, 30], which could lead to selection of more functional intersubtype. Based on this process, intersubtype recombination can often result in split codons (i.e. non-synonymous amino acid substitutions), insertions, deletions, and substitutions that can change the open reading frames and/or introduce premature stop codons.

In this study, we have screened out all of the intersubtype recombination events in *env* that obviously impact protein expression. Instead, we focused on the intersubtype recombination events with intact open reading frames that Env expression may or may not generate a functional chimeric protein. The HIV-1 *env* gene contains *tat2* and *rev2* exons that must be precisely spliced and joined to *tat1* and *rev1* exons to produce functional Tat and Rev proteins [26, 27]. There is a significant intersubtype sequence variability in both *tat* and *rev* sequences and there is evidence of co-evolution within the first and second exons of these accessory genes as well as within the TAR and RRE RNA target sequences [28, 29]. A recombination breakpoint in the gp120 coding sequence would result in an intersubtype discordance in *tat1* and *tat2* or *rev1* and *rev2*, which impact Tat and Rev expression as well as their function. Intersubtype A/D recombination in gp120 did not result in a frameshift or premature stop codon due to combination of discordant subtypes in the first and second exons. However, there was the possibility that discordant subtype sequences for the first and second exons of *tat/rev* impaired their function. In the present study, we found that two non-functional A/D *env* recombinants clones (out of 21) in an NL4-3 backbone were “rescued” for host cell entry and virus replication when “matching” *tat/rev* exons 1 and 2 were introduced. Interestingly, the other 19 A/D *env* recombinants clones in an NL4-3 backbone with the same functional Tat/Rev proteins remain non-functional, even though the Env glycoproteins were observed in all of these A/D recombinant viruses, suggesting that the discordance of *tat1/rev1* and *tat2/rev2* might not play the main role in restricting the function of resultant HIV-1 A/D intersubtype Env recombinants.

In the recombinant virus with surface expression of the A/D envelope glycoprotein, the lack of replication and host cell entry was most likely attributable to impaired function of this chimeric protein. Previous studies have described the possible non-covalent interactions between gp120 and gp41. The topology of the gp120 core structures implies that gp120 C1 and C5 are in close proximity [52–54], and immunological studies have also suggested interactions between the C1, C5, and the gp41 disulfide loop [23, 55]. The gp41 components are membrane-proximal and interspersed with the gp120 C1 and C5 elements. These two HR1 helices pack against hydrophobic gp120 residues in C1 and C5. The close proximity of C1 and C5 elements most likely participates in tertiary structure interactions with residues connecting HR1 to HR2 [40, 56]. The thermodynamic stability of this gp120–g41 interface is essential for the integrity of HIV-1 envelope trimer structure and function [40, 57]. The non-covalent nature of gp120-gp41 interaction presents significant challenges, for this contact must be of sufficient flexibility to maintain subunit interactions following gp120 binding to CD4 and subsequent gp120 conformational changes that promote its interaction with the CCR5 or CXCR4 co-receptor. The authenticity of this gp120–gp41 interaction for function is documented in experiments describing disruption due to minor temperature change, antibody or soluble CD4 binding, and inhibitor binding [57–59]. Based on these findings, we investigated if the expressed but non-functional Env was due to subtype discordance between the C1 and C5/ecto-gp41 domains introduced by intersubtype recombination in the gp120 coding region. First, cloning of the A/D gp120 coding region did not restore Env function or virus replication for any of the 9 selected clones in the NL4-3 backbone. However, the cloning of just V1-V5 regions resulted in a gain of function/replication for 4 of these 9 *envA/D* recombinant viruses. In this situation, the C1, C5/gp41 regions were retained from NL4-3 but V1-V5 gp120 sequences contained an upstream subtype A coding region fused to a subtype D coding region. Despite the extreme chimeric nature of the Env glycoprotein (e.g. subtype B-C1, A-V1 to V3, D-C4 to V5, B-C5 and B-gp41 for clone bk7169), many of these viruses with the A/D gp120 V1-V5 regions were functional. In contrast, a virus encoding the recombinant breakpoint as an Env cassette (e.g. subtype A–C1 to V3 and subtype D-C4 to gp41 for clone bk7169) could not mediate host cell entry and could not replicate despite having Env expression on the viral particle surface. Interestingly, Simon-Loriere et al found that defective intersubtype recombinants were most frequently observed in the C2 and C3 domains of C/B, A/C, and G/B *env* recombinants and less so in A/G and A/B *env* recombinants. Those nonfunctional intersubtype recombinants with C2 and C3 breakpoints may also be related to the co-evolution and interaction of the C1, C5 and gp41 domains [60].

The importance of this C1/C5/ecto-gp41 interaction for Env function is best supported by the recent 3 Å X-ray crystal structure of gp140 trimer in complex with a potent bnAb, PGT122 and the 5.8 Å cryo-electron microscopy re-construction of the Env trimer in complex with a CD4 binding site bnAb, PGV04 [36–38, 40–44]. In this study, we have utilized the available crystal structure coordinates to visualize this C1/C5/ecto-gp41 interaction within the gp140 trimer. As described in Fig. 7, the C1/C5/ecto-gp41 domains wrap together to form an inner core within the gp140 trimer. Thus, it is not surprising that major genetic shifts within this structure (e.g. intersubtype recombination events) may prevent proper Env function. This highly conserved structural motif may also be one of the reasons for the paucity of intersubtype recombination breakpoints in the gp120 coding region within the HIV-1 population but significant hot spots for recombination prior to the C1 region and following the ecto-gp41 domain of Env (http://www.hiv.lanl.gov/).

The close interaction between the C1/C5/ecto-gp41 domains within Env glycoprotein trimers suggests a possible co-evolution (co-variance) within these domains during HIV-1 divergence in the human epidemic. We specifically analyzed *env* sequences from a representative set of 144 and 97 unique HIV-1 subtype A1 and D isolates (http://www.hiv.lanl.gov) using two maximum likelihood/matrix models to test for co-evolution among gp120-C1,—C5, and ecto-gp41 domains. Based on these models, C1 and C5/ecto-gp41 domains had higher co-variance/co-evolution than any other paired gp120/gp41 regions (e.g. C1 + C2, C1 + C3, C1 + C4, etc.). Previous mutational analyses revealed evidences for C1, C5 and ecto-gp41 interactions. For example, V44A and F53A mutants can disrupt gp120/gp41 interactions [44] whereas gp120 mutations V36L and Y40D resulted in dissociation of gp120 from gp41 [61] despite interactions with cellular CD4 receptor [61]. C1 and C5 domains of gp120 form the two alpha helices that constitute the hydrophobic inner core that directly interacts with the immunodominant loop in the gp41 ectodomain [40, 42, 44]. Mutational studies revealed interactions between I491 of C5 and W596/S618 of the gp41 immunodominant loop [42, 62] and an additional A501 of C5 interacting with T605 of this gp41 loop [40]. Biochemical mapping of these C1/C5/ecto-gp41 interactions have now been confirmed with the recent gp140 trimer structure, which now suggests that many of these interactions are inter- as opposed to intramolecular interactions within the trimer C1/C5/ecto-gp41 core. Based on the present study, we cannot assume that breakpoints within the gp120 region would result in non-functional gp120 recombinants between other subtypes. However, with selection pressure for replication competency, we have rarely observed breakpoints within the gp120 of other intersubtype recombinants (http://www.hiv.lanl.gov/). Through analyzing 77 functional various intersubtype recombinants with single breakpoints in the *env* coding region, we have found that only 5 % have recombination sites in gp120. We are currently exploring the possible linked amino acids in the C1, C5 and ecto-gp41 domains that may interact in the gp140 trimer. As opposed to just structure-based mutagenesis studies, we can also identify key amino acid residues and structures in the trimer C1, C5 and ecto-gp41 core using a combination of co-evolution studies along with analyses of functional intersubtype recombinants in *env*.

## Conclusions

Studies on the gp120/gp41 structure of HIV-1 intersubtype Env recombinants could be the key to understanding the labile C1/C5/ecto-gp41 interface and could help in design of drugs to disrupt this interaction. Our study on HIV-1 *env* gene evolution in the epidemic reveals a strong link and co-evolution between the C1, C5 and ecto-gp41 domains which is less apparent between other subdomains of gp120 and gp41. These findings suggest a strong selective pressure for co-evolution in the C1, C5 and ecto-gp41 domains to maintain Env function and virus replication. In this study we have identified two mechanisms responsible for the genetic bottlenecks that select for specific replication-competent intersubtype Env recombinants. Of course, this was following the exclusion of the obvious that prevented Env expression, i.e. premature stop codons, insertions/deletions, and frameshifts. First, recombination within the gp120 coding region can generate non-functional or defective Rev and Tat proteins due to subtype/isolate discordance between the first and second exons of these accessory genes. Second, recombination within the gp120 coding region can introduce subtype/isolate discordance that disrupts the highly conserved C1/C5/ecto-gp41 interactions involved in the gp120/gp41 interface and critical for host cell entry.

## Methods

### HIV-1 primary isolates

A91 and A115 are HIV-1 subtype A strains, and D109 is HIV-1 subtype D strain, and they were all CCR5-tropic viruses. These viruses were collected from treatment-naive HIV-1-infected pediatric patients under IRB approval and patients consent through Joint Clinical Research Center (JCRC) in Kampala, Uganda in 1996. Neither patients nor their mothers had received any ARV treatment. The viruses were isolated and propagated by co-culturing Peripheral blood mononuclear cells (PBMCs) from the patients and from HIV seronegative blood donors as previously described [63]. PBMCs were obtained by Ficoll-Hypaque density gradient centrifugation of heparin-treated venous blood. Prior to HIV-1 infection, cells were stimulated with 2 μg of phytohemagglutinin (PHA) (Gibco BRL) per ml for 3 to 4 days and maintained in RPMI 1640– 2 mM L-glutamine medium (Cellgro) supplemented with 10 % fetal bovine serum (FBS, Cellgro), 1 ng of interleukin-2 (IL-2) (Gibco BRL) per ml, 100 U of penicillin (Cellgro) per ml, and 100 μg of streptomycin (Cellgro) per ml. TCID50 assays (tissue culture dose for 50 % infectivity) were performed to determine virus titer. Titers were expressed as infectious units per milliliter [64]. The sequence analysis of *env* genes of these three HIV-1 isolates were typical subtype A or D viruses.

### Cell lines

U87.CD4.CCR5 cell line was obtained from the AIDS Research and Reference Reagent Program and grown in Dulbecco’s modified Eagle’s medium (DMEM, Invitrogen) supplemented with 15 % FBS, penicillin and streptomycin, puromycin (1 μg/ml) and G418 sulfate (1 mg/ml). HEK-293 T cells were obtained from the American Type Culture Collection and grown in DMEM supplemented with 10 % FBS and penicillin and streptomycin. All cells were grown at 37 °C in 5 % CO2.

### Intersubtype recombination by dual infection

Production of intersubtype recombinant HIV-1 through in-vitro dual infection has previously been described [32]. One hundred and twenty thousand U87.CD4.CCR5 cells in a 12 well plate were inoculated with 0.1 multiplicity of infection (MOI) of each of primary HIV-1 isolate pair A91 and D109, or A115 and D109. Monoinfections were also set up as controls alongside the dual infections. The viruses were grown for 7 days before culture supernatant was harvested. HIV-1 viral RNA was extracted from the collected supernatant using the QIAamp Viral RNA mini kit (Qiagen), and was immediately used for Reverse transcription PCR (RT-PCR, see below).

### Reverse transcription PCR, PCR, and TOPO cloning

Viral RNA was extracted from 140 μL of culture supernatant using the QIAmpViral RNA mini kit (Qiagen), and was converted into cDNA by using Moloney murine leukemia virus RT (M-MLV, Invitrogen) and primer ENV-N (antisense, HXB2 numbering nt9146 to nt9172, 5′-CTGCCAATCAGGGAAGTAGCCTTGTGT-3′). Briefly, 5 μl of extracted viral RNA was mixed with 40 pmol of primer ENV-N, and cycled for 88 °C for 2 min, 70 °C for 10 min, 55 °C for 10 min, 37 °C for 10 min, and 4 °C hold. Next, 4 μl 5× first strand buffer (Invitrogen), 2 μl 0.1 M dTT (Invitrogen), and 1 μl 10 mM dNTPs were added to each reaction and cycled at 25 °C for 10 min, 42 °C for 2 min, and a 4 °C hold. Finally, M-MLV RT was added to the reaction and cycled at 42 °C for 1 h, 70 °C for 15 min, and a 4 °C hold.

To retrieve full length recombinant *envs* from the culture, nested PCR was performed with external primers ENV A (sense, nt5954 to nt5982, 5′-GGCTTAGGCATCTCCTATGGCAGGAAGAA-3′) and ENV M (antisense, nt9068 to nt9098, 5′- TAGCCCTTCCAGTCCCCCCTTTTCTTTTA-3′) followed by internal primers Vpu-A-1 (sense, nt6135 to nt6155, 5′-TAGTAGGTATAGAATATAAGA-3′) and gp41-D-2 (antisense, nt8747 to nt8769, 5′-GCCTAATTCTTCTAGGTATGTTG-3′). PCR amplifications were performed with the following conditions: 94 °C 4 min, [94 °C 30 s, 55 °C 30 s, 72 °C 3 min] × 35 cycles, and 4 °C hold. Please note, in order to eliminate the possible PCR-induced *env* recombinants, we used Phusion High-Fidelity DNA Polymerase (NEB) which can rapidly complete the DNA extension (15–30 s per Kb) and longer extension time (3 min) than the requested 1.3 min. PCR products (~2.6 kb) of HIV-1 recombinant *envs* were gel purified with a QIAquick gel extraction kit (Qiagen), and were cloned into TOPO-TA PCR cloning vector (Invitrogen) which was then transformed into OneShot Top10 *E. coli* cells (Invitrogen) according to manufacturer’s instructions. The transformed cells were plated onto LB (Luria-Bertani) plates containing 100 μg/ml ampicillin, X-gal and Isopropyl β-D-1-thiogalactopyranoside (IPTG). The yielded clones were grown for plasmid extraction using a QIAamp plasmid miniprep kit (Qiagen) and the resultant DNA was sent for clonal sequencing. Full-length and V1-V5 domains of recombinant *gp120* were amplified from TOPO vectors using sense primer VPU-A-1, and different antisense primers gp120-D-2 (nt7617 to nt7642, 5′- CCTCCTCCAGGTCTGAAGGTTTCATT-3′) and V5-D-2 (nt7373 to nt7399, 5′-TTAAACATATGTGTTGTAATCTCTAA-3′) respectively. To further exclude the possible recombinants generated by the Taq polymerase jumping between the templates, we set up a control PCR by using the mixed proviral DNAs from the monoinfections with subtype A or D isolates. The results showed that the frequency of these background recombinants was always lower than 0.5 %, or at least 20- fold less than the HIV-1 recombination frequency obtained with heterozygous virions.

### Cloning of full length recombinant *envs* and *gp120s* into NL4-3 backbone

The pREC_nfl_HIV_NL4-3_ vector containing a near full length (nfl) HIV-1 isolate NL4-3 backbone has previously been described [34] and routinely used to clone different HIV genes to create various chimeric HIV-1 viruses through yeast gap repair homologous recombination [2, 65, 66]. To clone an HIV-1 sequence into pREC_nfl_HIV_NL4-3_ vector, the corresponding region in the NL4-3 backbone is first replaced with the yeast orotidine 5-phosphate decarboxylase (*URA3*) gene using specific primers tagged with 40–60 nucleotides to allow for homologous recombination at either side of the target sequence. We created cloning shuttle plasmids pREC_nfl_HIV∆env/URA3, pREC_nfl_HIV∆gp120/URA3, pREC_nfl_HIV∆V1-V5/URA3 and pREC_nfl_HIV_envA/D_∆tat1/URA3 for cloning full length recombinant HIV-1 *env*, *gp120*, gp120 *V1-V5* domains and D109 *tat1* respectively. To clone the URA3 products into the NL4-3 backbone to create the corresponding shuttle plasmids, The *Saccharomyces Cerevisiae* yeast MYA-906 (genotype *MATα-ade6-can1-his3-leu2-trp1-URA3*) was grown in 5 ml YEPD media at 30 °C on a shaking platform overnight. After 12–16 h, 0.5 ml of the yeast culture was inoculated in 50 ml YEPD media and grown for 4–6 h to attain an OD600 of 1.0. The yeast was washed in 1 ml of sterile water and resuspended in 1 ml of fresh 1× LiAc/TE solution in water [LiAc (100 mM pH 7.5) and TE (10 mM Tris-Cl pH 7.5 and 1 mM EDTA pH 8.0)] to induce competence and then chilled on ice. The PCR inserts (3 μg) and the linearized pREC_nfl_HIV_NL4-3_ plasmid (1 μg) were then co-transformed along with 50 μg of denatured salmon sperm carrier DNA (BD Biosciences/Clontech, Palo Alto, CA) into yeast using the lithium acetate-PEG. The mixture was incubated on a shaking platform at 30 °C for 30 min followed by a 15 min heat shock at 42 °C and then plated on complete supplement media (CSM)-LEU-URA agar plates in which only the yeast containing successful replacement of certain HIV sequences (i.e. *env*, *gp120*, *V1-V5*, or *tat1/env*) by *URA3* gene can grow. Plasmids were retrieved from yeast clones using a mixture of mechanical glass bead disruption and phenol-chloroform-IAA extraction, and 4 μL of the crude yeast preparation was transformed into STBL4 *E.coli* (Invitrogen). The shuttle plasmids pREC_nfl_HIV∆env/URA3, pREC_nfl_HIV∆gp120/URA3, pREC_nfl_HIV∆V1-V5/URA3 and pREC_nfl_HIV_envA/D_∆tat1/URA3 were finally extracted from STBl4 *E. coli* and were used for the subsequent experimentation.

To clone the different PCR products into their corresponding shuttle plasmids, the same yeast transformation procedure was performed, but with different shuttle vectors and selected on CMM-Leu + 5-fluoro-1,2,3,6-tetrahydro-2,6-dioxo-4-pyrimidine carboxylic acid (5-FOA) plates in which only the yeast containing the shuttle vector with successful replacement of *URA3* by the corresponding HIV-1 *env* (or *gp120*, *V1-V5*, and *tat1/env*) recombinants can survive and generate pREC_nfl_HIVenvA/D, pREC_nfl_HIVgp120A/D, pREC_nfl_HIV_V1-V5A/D and pREC_nfl_HIVtat1/envA/D.

### Veritrop assay

One million U87.CD4.CCR5 cells were plated in 100 mm petri dishes (Day 1), and 24 h later (Day 2), were transfected with pDM.128-fLuc plasmid using FuGENE 6 transfection reagent (Roche). On day 1 also, 6.5 × 10^4^ HEK-293 T cells (per well) were plated in 24-well plate, and 24 h later (Day 2), were transfected with pREC_nfl_HIV plasmid containing the respective *env* recombinant inserts. Six hours post-transfection, U87.CD4.CCR5 cells were plated into a 24-well plate at 6.5 × 10^4^ cells per well. On day 3, transfected HEK-293 T cells from each well were collected and 6.5 × 10^4^ cells were layered over the seated 87.CD4.CCR5 cells and co-cultured for an additional 15 h. Lastly, the cells were lysed with 100 μl of Glo Lysis Buffer for 15 min at room temperature, and then 50 μl of lysate was mixed with 50 μl of Bright Glo (Promega Biotech) and luminescence was read on Victor^3^ V (Perkin Elmer) luminometer for luciferase activity.

### Virus production

Production of infectious HIV-1 from pREC_nfl_HIVenvA/D (or pREC_nfl_HIVgp120A/D, pREC_nfl_HIV_V1-V5A/D, and pREC_nfl_HIVtat1/envA/D) vectors containing various A/D *env* recombinants were done according to Dudley et al. [34]. Briefly, on day 1, 7 × 10^4^ HEK-293 T cells/well were plated, and 24 h later (Day 2), 0.3 μg of each of the pREC_nfl_HIVenvA/D plasmids containing various HIV-1 A/D *env* recombinant sequences were co-transfected with an equal amount of a complementing plasmid pREC_5′LTR_gag/pol (containing only the R, U5, PBS, uncoding regions, gag, and partial pol sequences) [67]. The next day (day 3) 7 × 10^4^ U87.CD4.CCR5 cells were plated in a 24 well plate, and infected 24 h later (Day 4) by transfer of the supernatant from the transfected HEK-293 T cells. After 24 h, infection supernatant was washed off and the U87.CD4.CCR5 cell cultures maintained for up to 2 weeks following infection. Virus replications in U87.CD4.CCR5 cell culture were monitored by visually monitoring syncytia appearance and measuring reverse transcriptase activity every 2–3 days. Supernatant from infected wells was harvested and frozen at—80 °C for future use.

### Reverse transcriptase-activity assay (RT Assay)

Ten microliter of cell culture supernatant was collected into a 96-well plate every other day and subjected to RT activity assay as previously described [68]. Briefly, 10 μl of transfected and/or infected supernatant was incubated at 37 °C overnight in the presence of 25 μl RT mixture (1 μl of fresh 10 mCi/mL [α-32P]-dTTP in 1 ml RT master mix: 50 mM Tris-HCl (pH 7.8), 75 mM KCl, 2 mM DTT, 5 mM MgCl_2_, 5 μg/ml of poly(rA), 6.25 μg/ml oligo(dT), and 0.5 % (v/v) NP40). After incubation, 10 μl of the reaction mixture was blotted from each well onto the 96-well format DEAE filtermat. After 10 min of drying, the filtermat was washed five times with 1 × SSC solution and twice with 85 % ethanol (5 min for each time) in a shaking platform. The filters were then wrapped with Saran wrap and either exposed overnight onto autoradiography film (Kodak BioMax MR) or counted with a Matrix 96 β-counter (Packard, Meriden, CT) to acquire the quantitative RT activity.

### Clonal sequencing

We procured commercial sequencing services of ACGT, Inc (Wheeling, Illinois). Full length *envs* were sequenced using five universal primers Vpu-1 (nt6108–6130; sense, 5′-TAATAATAGCAATAGTTGTGTGG-3′), E80 (nt6862-6883; sense, 5′-TTCCAATACACTATTGTGCTCC-3′), EAD2 (nt8064–8084; antisense, 5′-CCAGAGATTTATTACTCCA-3′), E15 (nt8424–8425; antisense, 5′-CTTGCTCTCCACCTTCTTCTTC-3′) and ENVM (nt9068–9096; antisense, 5′-TAGCCCTTCCAGTCCCCCCTTTTCTTTTA-3′). Sequencing chromatographs were physically inspected to ensure accuracy of sequencing data, and fragments were joined to form full length *envs* and aligned along with primary *envs* of isolates A91, A115, and D109 with BioEdit Sequence analysis software. Aligned sequences were imported into SimPlot sequence similarity plotting software and bootstrap plots were constructed with the recombinant sequences as the query and the two recombining primary *envs* as the references. The Kimura-2 parameter model was used for analysis with a 200 and 20 base pair window and step respectively.

### In-house ELISA

Costar 96-well EIA/RIA plates (Immunochemistry technologies) were coated with 100 ng/well of concentrated pseudoviruses or with commercially obtained recombinant HIV-1 gp140 (UG37) (as a positive control). Detection of envelope expression on pseudoviruses was done using of b13 monoclonal antibody (provided by George Lewis, Institute of Human Virology, Baltimore, MD, and Bruce Chesebro, NIAID, Hamilton, MT), followed by addition of rabbit anti-mouse IgG-HRP conjugate antibody (Promega Cat#W4021) at a dilution of 1:2500, and TMB substrate. The reaction was stopped with 1 N sulfuric acid, the developed chromogen was read at λ = 450 nm, and the optical density (OD) was plotted.

### Western blot

HIV-1 viral Env proteins were detected by Western blot. Blots were performed on virus pelleted from the culture supernatants. Virus-containing supernatants were clarified of cellular debris by centrifugation (2500 rpm, 15 min) and virions were pelleted by centrifugation at 32,000 rpm for 1 h. Samples were resuspended in sodium dodecyl sulfate (SDS) lysis buffer (40 mM Tris-HCl [pH 6.8], 10 % glycerol, 10 % β-mercaptoethanol, 1 % SDS) and heated to 95 °C for 5 min, separated by SDS-10 % polyacrylamide gel electrophoresis, and transferred to nitrocellulose. Membranes were blocked with 5 % milk, incubated with the B13 antibody and then with horseradish peroxidase-conjugated secondary antibodies, detected with the ECL Plus kit (Amersham Biosciences, Piscataway, NJ), and finally exposed to x-ray film.

### Phylogenetic/coevolution analyses

Intra-protein coevolution may be inferred by quantifying the degree of similarity of phylogenetic tree structure of two protein domains based on the correlation of the distance matrices used to build the trees [47]. To measure the covariance between the conserved regions of gp120 and the gp41 extracellular domain, full length subtype A1 and subtype D envelope amino acid sequences were downloaded from the Los Alamos National Laboratory HIV sequence database (http://www.hiv.lanl.gov/). The sequences were filtered to include one sequence per patient and sequences with more than one ambiguous codon were removed, which resulted in data sets containing 144 and 97 unique sequences, respectively. Subtype specific alignments were performed using MUSCLE [69] then trimmed to the positions containing the C1–C5 regions and the ecto-gp41 domain (Fig. 8a). Maximum likelihood trees were generated for each region using the algorithms implemented in PHYML [70] with 100 bootstrap replicates, and the tree topology search was performed using Nearest Neighbor Interchanges (NNIs). The phylogeny of the regions was estimated using the rtREV [45] model of amino acid substitution, which was developed specifically for phylogenetic inference of retroviral sequence. The equilibrium frequencies and the gamma distribution were both estimated by the model, and the number of substitution rate categories was set to 4. The resulting maximum likelihood trees were used as input to the Mirrortree web server [46] which was developed to predict functional relationships between protein families based on the tree topology of the proteins or domains being compared (Fig. 8b). This algorithm implements the tree correlation method developed by Pazos and Valencia [47] that creates a distance matrix for each tree by summing the branch lengths that separate the taxa in the tree, and then estimating the linear correlation coefficient (r) between the two matrices. Heatmaps demonstrating the correlation between each region in *env* were produced with Plot (https://plot.ly).

Additionally, pairwise distance matrices of each region were built with MEGA [71], which used a Jones-Taylor-Thornton (JTT) matrix-based model [48] and a uniform substitution rate among sites, to calculate the number of amino acid substitutions per site/residue from between each sequence in the alignment. Correlation between each conserved region in gp120 or the gp41 domain were then measured by Pearson’s product-moment correlation coefficient, and significance estimated with Fisher’s exact test (Fig. 8c).

## Abbreviations

HIV-1: Human immunodeficiency virus type 1
URF: Unique recombinant form
CRF: Circulating recombinant form
